# Efficacy and brain modulation mechanisms of acupuncture for chronic prostatitis/chronic pelvic pain syndrome revealed by structural MRI changes

**DOI:** 10.3389/fneur.2025.1579484

**Published:** 2025-06-25

**Authors:** Zhen Wang, Jinmei Wu, Cong Deng, Yongbo Hu, Songshan Shi, Shaowei Liu, Xinfei Huang, Jianhuai Chen

**Affiliations:** ^1^Department of Acupuncture, Foshan Hospital of Traditional Chinese Medicine (Gaoming Branch), Foshan, China; ^2^Department of Traditional Chinese Medicine, Foshan Hospital of Traditional Chinese Medicine (Gaoming Branch), Foshan, China; ^3^Department of Urology, Nanjing Integrated Traditional Chinese and Western Medicine Hospital Affiliated with Nanjing University of Chinese Medicine, Nanjing, China; ^4^Department of Radiology, Jiangsu Province Hospital of Chinese Medicine, Affiliated Hospital of Nanjing University of Chinese Medicine, Nanjing, China; ^5^Department of Andrology, Nanjing Hospital of Traditional Chinese Medicine, Nanjing Hospital of Chinese Medicine Affiliated to Nanjing University of Chinese Medicine, Nanjing, China; ^6^Department of Andrology, Jiangsu Province Hospital of Chinese Medicine, Affiliated Hospital of Nanjing University of Chinese Medicine, Nanjing, China

**Keywords:** chronic prostatitis/chronic pelvic pain syndrome, acupuncture, National Institute of Health-Chronic Prostatitis Symptom Index, structural magnetic resonance imaging, gray matter, white matter

## Abstract

**Introduction:**

Chronic prostatitis/chronic pelvic pain syndrome (CP/CPPS) has been considered to be associated with abnormal brain function and structure. Acupuncture is a promising therapy for CP/CPPS, however, the underlying brain modulation mechanisms of acupuncture for CP/CPPS are still unclear.

**Methods:**

A total of 25 CP/CPPS patients and 25 matched-healthy controls (HCs) were enrolled. All patients received acupuncture treatment 3 times weekly for 4 weeks with a total of 12 sessions [acupoints including Guanyuan (RN4), Zhongji (RN3), Zusanli (LR10), Zuwuli (ST36), Sanyinjiao (SP6), and Yinlingquan (SP9)]. The efficacy was evaluated by the National Institute of Health-Chronic Prostatitis Symptom Index (NIH-CPSI). In addition, structural T1-weighted magnetic resonance imaging (MRI) brain scans were acquired from all patients before and after treatment, as well as HCs. MRI data were preprocessed and the measures of gray matter volume and density, as well as white matter volume and density, were calculated for all subjects. Finally, all these measures were compared between patients (before and after treatment) and HCs, and were also compared within patients before and after treatment. Moreover, the relationships between brain structure and NIH-CPSI scores were also evaluated.

**Results:**

After treatment, CP/CPPS patients demonstrated decreased scores in the scale of NIH-CPSI and its subscales. Compared with HCs, both CP/CPPS patients before and after treatment showed increased gray matter volume and density, as well as increased white matter volume and density, especially in the frontal and parietal regions. After treatment, CP/CPPS patients showed decreased gray matter volume in the left middle cingulate gyrus, as well as increased gray matter volume and density in the left middle occipital gyrus. In addition, these structural brain abnormalities were related to NIH-CPSI scores of patients while the changes of NIH-CPSI scores were associated with the changes of structural changes in the brain of CP/CPPS patients before and after treatment.

**Conclusion:**

These findings suggested that the development of CP/CPPS might be associated with the increased gray matter and white matter in the frontal, cingulate and parietal regions. The effects of acupuncture in improving clinical symptoms of CP/CPPS might be achieved by reducing the gray matter volume in the left middle cingulate gyrus.

## 1 Introduction

Prostatitis is a common disorder affecting nearly 10–14% of adult men, and the overall lifetime prevalence increases with age (1, 2). Approximately 27% of men suffer from prostatitis at least once a year and about 16% of men experience persistent symptoms of prostatitis (1). According to the National Institutes of Health (NIH) classification, prostatitis is divided into four types: type I/II, acute/chronic bacterial prostatitis; type III, chronic prostatitis/chronic pelvic pain syndrome (CP/CPPS) and type IV, asymptomatic prostatitis (3). The most common form is CP/CPPS and it is subclassified into type IIIa and IIIb (inflammatory and non-inflammatory) according to the presence or absence of leukocytes in expressed prostatic secretion (EPS) (4). CP/CPPS is diagnosed when the pelvic pain is present for at least 3 months of the preceding 6 months in the absence of a urinary tract infection or other identifiable causes (5). CP/CPPS patients often present with a wide variety of clinical signs and symptoms, including pelvic pain, lower urinary tract symptoms (LUTS), psychological issues and sexual dysfunction (6). However, the details of pathological mechanisms underlying CP/CPPS are unclear and the current clinical treatments for CP/CPPS mainly focus on the symptomatic treatment. In addition, the mechanisms underlying the therapeutic efficacy of the treatments, especially non-pharmacological treatments, remain largely unclear.

Many different etiologies of CP/CPPS have been proposed, including local inflammation or cryptic infections, defective urothelial integrity and function, pelvic floor muscle irregularities, voiding dysfunction, endocrine imbalances, autoimmunity, peripheral and central sensitization and neuroplasticity, as well as psychosocial factors (7–13). In addition, CP/CPPS is regard as a kind of chronic pain disorder and patients often manifest various psychiatric and/or psychosocial symptoms (14, 15). The brain plays a key role in the regulation of pain and emotion (16–18). In addition, both functional and structural brain alterations have been identified in patients with chronic pain and emotional disorders (19, 20). Therefore, it was speculated that CP/CPPS might also result from the abnormal central processes and altered brain activity, as well as impaired brain structure associated with CP/CPPS have been characterized in previous neuroimaging studies (11, 21–23). In our previous study (8), we explored its central pathological mechanisms by evaluating the topological changes of white matter brain networks in patients with CP/CPPS based on diffusion tensor imaging data. CP/CPPS showed lower global efficiency in the right orbital middle frontal gyrus, higher global efficiency in the left middle cingulate and paracingulate gyrus, as well as increased local efficiency in the left middle cingulate, paracingulate gyrus and paracentral lobule (8). Moreover, the changed topological measures of left middle cingulate gyrus, left precuneus and right supplementary motor area were correlated with the scores of National Institute of Health-Chronic Prostatitis Symptom Index (NIH-CPSI). These findings implied that the altered brain structure might contribute to the pathogenesis of CP/CPPS.

The pharmacological or non-pharmacological treatments for CP/CPPS include antibiotics, α-adrenergic antagonists, non-steroidal anti-inflammatory drugs (NSAIDs), phytotherapy and acupuncture, which are merely symptomatic (2, 6, 24–27). Acupuncture, one of the traditional Chinese medicine (TCM) therapies, which has been recommended by the 2025 European Association of Urology guidelines on chronic pelvic pain, is considered as an effective treatment for CP/CPPS, particularly in relieving pain (28). A previous systematic review included randomized controlled trials (RCTs) comparing acupuncture with sham acupuncture/medication or comparing acupuncture and medication with the same medication, which showed that acupuncture was beneficial in treating CP/CPPS, especially in reducing the NIH-CPSI total and pain scores (29). Previous meta-analysis demonstrated that acupuncture has measurable benefits on CP/CPPS (pain, urinary symptom, quality of life, NIH-CPSI score, and efficacy rate), and the security of acupuncture was also ensured (30). Magnetic resonance imaging (MRI), especially functional MRI (fMRI), is a novel method for exploring the changes of brain activity associated with acupuncture treatment (31). Previous systematic review study showed that acupuncture at ST36 could activate the opercular part of the right inferior frontal gyrus, left superior temporal gyrus and right median cingulate/paracingulate gyri regions (32). In addition, previous fMRI study implied that acupuncture could concurrently regulate the resting state functional connectivity of two pain modulation regions in the amygdala and middle cingulate cortex, which were be the key regions linked to multisensory processing of pain modulation in chronic pain with acupuncture treatment (33).

Therefore, it is important to identify the acupuncture-stimulated brain regions of CP/CPPS patients, which may help to elucidate the neural mechanisms of acupuncture in treating CP/CPPS. This study aimed to explore the underlying brain modulation mechanisms of acupuncture for CP/CPPS. In this study, structural MRI data were acquired from CP/CPPS patients before and after receiving acupuncture treatment. The efficacy was evaluated by the NIH-CPSI and the changes of brain structure associated with acupuncture were measured by gray matter volume and density, as well as white matter volume and density. In addition, the relationships between brain structure and NIH-CPSI scores were evaluated in CP/CPPS patients.

## 2 Materials and methods

### 2.1 Participants

All consenting procedures and protocols of the current study were approved by the ethics committee of Jiangsu Province Hospital of Chinese Medicine. All participants provided informed written consents to participate in this study. A cohort of 25 CP/CPPS patients and 25 matched-healthy controls (HCs) were recruited. According to the National Institutes of Health definition of CP/CPPS, the diagnosis of CP/CPPS was based on the chief complaint, history-taking, physical examination, laboratory examination including routine urine and standard microbiological cultures of urine, prostate and urinary system ultrasound. Based on these examinations and tests, acute or chronic bacterial prostatitis, benign prostate hyperplasia and other related diseases among the pelvic were excluded.

All participants included in the study were Han Chinese, right-handed and aged from 20 to 60 with no less than 9 years of education. Inclusion criteria for CP/CPPS participants consisted of: (1) met the criteria for the diagnosis of CP/CPPS according to the NIH criteria (34); (2) all patients were type IIIb (non-inflammatory) in the absence of leukocytes in EPS; (3) had complaints about pelvic pain lasting for at least 3 months of the preceding 6 months in the absence of a urinary tract infection or other identifiable causes; (4) all patients had total NIH-CPSI scores ≥ 15 and NIH-CPSI pain sub-score ≥ 6. In addition, HCs had no chronic pain in pelvic region, and chronic pain in other body region.

The exclusion criteria for all participants consisted of the following: (1) accompanied by other urologic diseases, such as urinary tract infection, acute or chronic bacterial prostatitis, epididymitis, benign prostatic hyperplasia (BPH), urinary stones, groin hernia, varicocele; (2) history of surgery, deformity or cancer in the genitourinary system; (3) pelvic pain caused by the colorectal or lumbar diseases, or accompanied by chronic pain in other body sites; (4) history of psychiatric, neurological disorders, other serious or acute physical illness; (5) substance abuse or dependence; (6) treatment with painkillers, antidepressants or other psychotropic medications affecting brain function and structure within 3 month prior to enrollment; (7) visible brain abnormalities in conventional T1- and T2-weighted images; (8) any contraindication to MRI.

### 2.2 Acupuncture intervention

The protocol consisted of a standardized set of acupuncture points given 3 times weekly for 30 min over 4 weeks with a total of 12 sessions. The acupuncture points were selected as Guanyuan (RN4), Zhongji (RN3), Zusanli (LR10), Zuwuli (ST36), Sanyinjiao (SP6), and Yinlingquan (SP9).

Acupuncture procedure: (1) instruct the patient to take a supine position, keep the whole body relaxed, and perform routine disinfection; (2) acupuncture at Guanyuan point (after urination), using a 1.5-inch needle to directly puncture 1–1.5 inches; (3) acupuncture at Zhongji point (after urination), using a 1.5-inch needle to directly puncture 1–1.5 inches; (4) acupuncture the Zusanli point, using a 1.5-inch needle to directly puncture 1–2 inches; (5) acupuncture the Zuwuli point (avoiding the artery), using a 1.5-inch needle to directly puncture 1–1.5 inches; (6) acupuncture at Sanyinjiao acupoint, using a 1.5-inch needle to directly prick 1–1.5 inches; (7) acupuncture the Yinlingquan acupoint, using a 1.5-inch needle to directly prick 1–1.5 inches.

The needle was retained for 30 min, and the first, 15, and 30 min were, respectively, injected once, three times in total. The acupuncture manipulation of lifting, inserting, twisting, and rotating for reinforcing and reducing was used, of which Guanyuan and Zusanli points were used for reinforcing, Zhongji and Zuwuli points were used for reducing, and Sanyinjiao and Yinlingquan points were used for reinforcing and reducing.

### 2.3 Questionnaires

The NIH-CPSI scale was applied to assess the severity of CP/CPPS symptoms (35). The NIH-CPSI has a total of 9 items divided into 3 domains as follows: (1) pain or discomfort including the location, type, severity and frequency (4 items, 0–21 points); (2) urinary symptoms including irritative and obstructive urinary symptoms (2 items, 0–10 points); (3) quality-of-life (QoL) (3 items, 0–12 points). The higher total score (0–43 points) and higher three sub-scores indicate more severe symptoms. All of the patients were requested to finish NIH-CPSI scale before and after acupuncture intervention.

### 2.4 MRI data acquisition and preprocessing

Structural MRI data of all participants were acquired using a 3.0 T Siemens MRI scanner. The detailed scanning parameters were the same as those in our previous study (36). Preprocessing of Structural MRI was carried out using the software of Data Processing Assistant for Resting-State fMRI (DPARSF) (37) based on MATLAB. The preprocessing steps, and the calculation of gray matter volume and density, as well as white matter volume and density, have been elucidated in previous studies (38–42).

### 2.5 Statistical analysis

By the software of Statistical Package for the Social Sciences (SPSS), *t*-tests were used for data conforming to normal distribution, while non-parametric tests were used for data not conforming to normal distribution, to evaluate the differences of demographic data between CP/CPPS patients and HCs, as well as the changes of NIH-CPSI total scores and sub-scores in patients before and after acupuncture intervention. *p* < 0.05 was considered to be statistically significant difference.

In addition, two sample *t*-tests were performed to evaluate the differences of gray matter volume and density, as well as white matter volume and density between CP/CPPS patients (before and after treatment) and HCs using the software of Resting-State fMRI Data Analysis Toolkit (REST) (43). Moreover, paired-sample *t*-tests were used to evaluate the changes of gray matter volume and density, as well as white matter volume and density in patients before and after treatment. The significant difference was set at *p* < 0.001 and the minimum cluster size was 6 voxels, which was corrected by the AlphaSim program in REST software. Finally, the relationships between NIH-CPSI scores and brain structure in CP/CPPS patients were explored by *Pearson* correlation analysis. *p* < 0.05 was considered to be statistically significant difference.

## 3 Results

### 3.1 Demographic and clinical data of CP/CPPS patients

There were no significant differences in the age and educational level between CP/CPPS patients and HCs. When compared with patients before treatment, CP/CPPS patients after treatment demonstrated decreased NIH-CPSI scores including total scores, pain or discomfort scores, symptom severity scores and quality-of-life impact scores. CP/CPPS patients after treatment demonstrated no significant differences in urinary symptom scores when compared with patients before treatment (Table 1).

**Table 1:** Demographic and clinical characteristics of CP/CPPS patients.

Characteristics	CP/CPPS	HCs	*t*/*Z*	*P*
Age (years)	31.76 ± 9.68	32.28 ± 7.23	−0.22	0.83^a^
Educational level (years)	14.04 ± 3.26	14.44 ± 1.64	−0.41	0.68^b^
NIH-CPSI (before/after treatment)				
Total scores	25.40 ± 5.41/16.84 ± 3.02	–	−12.10	<0.01^a^
Pain or discomfort scores	12.92 ± 2.71/7.00 ± 1.26	–	−5.90	<0.01^b^
Urinary symptom scores	3.48 ± 2.90/2.96 ± 2.13	–	−0.40	0.69^b^
Symptom severity scores	16.40 ± 4.00/9.96 ± 2.62	–	−5.17	<0.01^b^
Quality-of-life impact scores	8.88 ± 2.17/6.96 ± 1.06	–	−3.27	<0.01^b^

CP/CPPS, chronic prostatitis/chronic pelvic pain syndrome; NIH-CPSI, National Institute of Health-Chronic Prostatitis Symptom Index.

a *p* values were obtained by *t*-tests.

b *p* values were obtained by non-parametric tests.

*P* < 0.05 was considered to be statistically significant difference.

### 3.2 Abnormal gray matter volume and density in CP/CPPS patients before treatment compared with HCs

**Figure 1:**
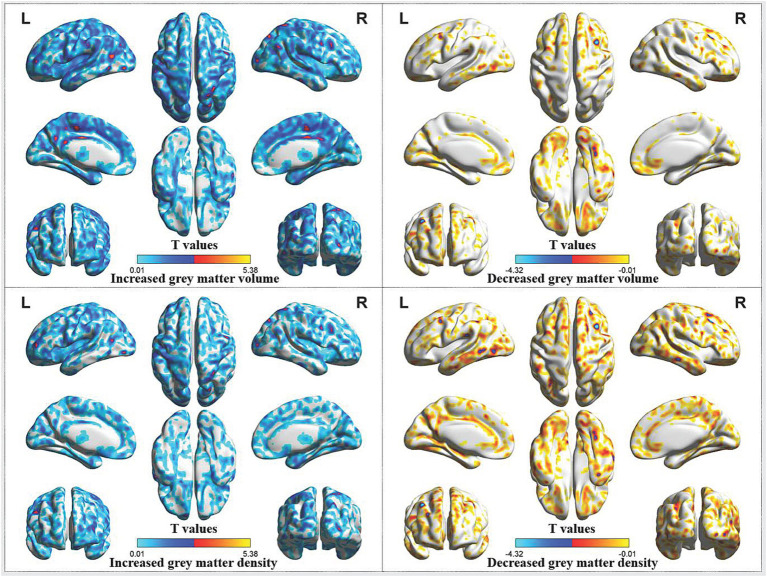
Abnormal gray matter volume and density in CP/CPPS patients before treatment compared with HCs. CP/CPPS, chronic prostatitis/chronic pelvic pain syndrome; HCs, healthy controls; L, left; R, right.

Compared with HCs, CP/CPPS patients before treatment showed increased gray matter volume in the left middle cingulate gyrus, left precentral gyrus, left middle temporal gyrus, left middle occipital gyrus, right middle cingulate gyrus and right superior parietal gyrus, as well as decreased gray matter volume in the right middle frontal gyrus. These patients also exhibited increased gray matter density in the left precentral gyrus, decreased gray matter density in the right middle frontal gyrus and right middle occipital gyrus (Table 1; Figure 1).

The functions of the frontal lobe are mainly related to mind, language, and voluntary movement, while the functions of the parietal lobe are mainly related to sensation and cognition. The functions of cingulate gyrus are very complex, mainly including the following aspects: emotional regulation, cognitive control, pain management, and social behavior. The above findings suggested that the development of CP/CPPS might be associated with the increased gray matter in the frontal, cingulate and parietal regions, which might be associated with the clinical manifestations of CP/CPPS patients.

### 3.3 Abnormal white matter volume and density in CP/CPPS patients before treatment compared with HCs

**Figure 2:**
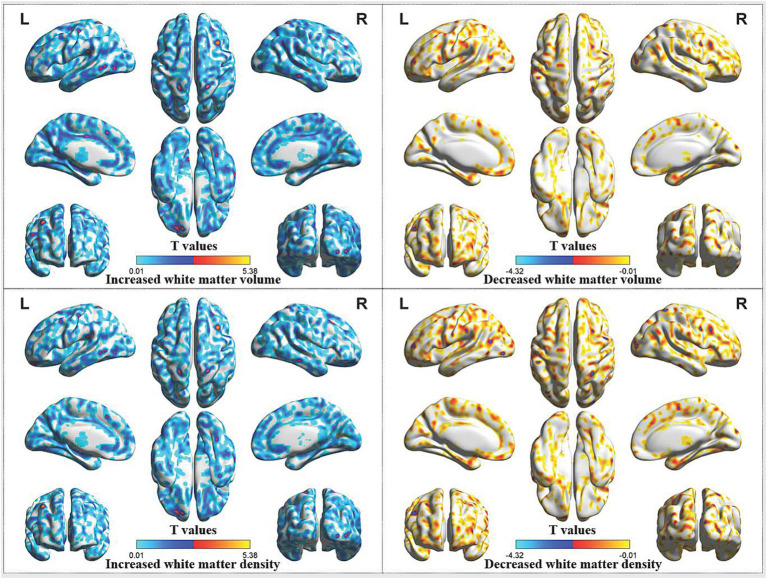
Abnormal white matter volume and density in CP/CPPS patients before treatment compared with HCs. CP/CPPS, chronic prostatitis/chronic pelvic pain syndrome; HCs, healthy controls; L, left; R, right.

In comparison with HCs, increased white matter volume were identified in the left orbital superior frontal gyrus, left medial superior frontal gyrus, left superior parietal gyrus, left postcentral gyrus, left inferior temporal gyrus, left supramarginal gyrus, right middle frontal gyrus, right postcentral gyrus, right middle temporal gyrus, right middle occipital gyrus and right angular gyrus of CP/CPPS patients before treatment. In addition, patients before treatment showed increased white matter density in the left orbital superior frontal gyrus, left superior parietal gyrus, left postcentral gyrus, left supramarginal gyrus, right middle frontal gyrus, right middle occipital gyrus and right postcentral gyrus, as well as decreased white matter density in the left precentral gyrus (Table 2; Figure 2).

**Table 2:** Brain regions exhibited abnormal gray matter volume and density in CP/CPPS patients.

Brain regions	Peak MNI coordinates			Clusters	Peak *t* values
	*x*	*y*	*z*		
Gray matter volume					
Patients before treatment vs HCs					
Left middle cingulate gyrus	−4	−16	32	57	4.38
Left precentral gyrus	−36	2	42	10	5.38
Left middle temporal gyrus	−48	−64	16	21	4.41
Left middle occipital gyrus	−42	−80	−2	7	4.19
Right middle cingulate gyrus	6	−38	38	36	4.31
Right superior parietal gyrus	28	−48	60	9	4.74
Right middle frontal gyrus	36	20	44	14	−4.32
Patients after treatment vs HCs					
Left middle cingulate gyrus	−2	−28	32	17	4.27
Left precentral gyrus	−36	2	42	10	5.34
Left middle temporal gyrus	−48	−64	16	19	4.44
Left middle occipital gyrus	−42	−82	−2	9	4.32
Left putamen	−24	−2	2	21	3.77
Right middle cingulate gyrus^1^	6	−38	38	33	4.33
Right middle cingulate gyrus^2^	6	−22	42	28	4.14
Right superior parietal gyrus	28	−48	60	8	4.75
Right postcentral gyrus	18	−38	80	7	3.87
Right parahippocampal gyrus	26	−2	−36	10	3.99
Right middle frontal gyrus	34	20	44	12	−4.23
Patients after vs before treatment					
Left middle cingulate gyrus	−6	−2	38	7	−4.03
Left middle occipital gyrus	−28	−94	20	9	4.77
Gray matter density					
Patients before treatment vs HCs					
Left precentral gyrus	−36	2	44	9	5.51
Right middle frontal gyrus	36	20	44	21	−4.61
Right middle occipital gyrus	36	−78	2	9	−4.30
Patients after treatment vs HCs					
Left precentral gyrus	−36	2	44	9	5.44
Left inferior occipital gyrus	−44	−80	−4	7	4.23
Right middle frontal gyrus	36	20	44	20	−4.48
Right middle occipital gyrus	36	−78	2	7	−4.28
Patients after vs before treatment					
Left middle occipital gyrus	−28	−94	20	6	4.14

CP/CPPS, chronic prostatitis/chronic pelvic pain syndrome; MNI, Montreal Neurological Institute; x, y, and z, the coordinates of peak voxels of clusters in MNI space. The significant difference was set at *P* < 0.001 and the minimum cluster size was 6 voxels, which was corrected by the AlphaSim program in REST software. ^1^Cluster 1 in right middle cingulate gyrus. ^2^Cluster 2 in right middle cingulate gyrus.

The frontal and parietal regions also showed increased white matter, which might be also related to the development of CP/CPPS. The increase in white matter of in the frontal and parietal lobes might be a compensatory mechanism, which might increase sensitivity to urgency of urination and pelvic pain in CP/CPPS patients. On the contrary, it might also be the result of compensatory increase of brain structure caused by LUTS symptoms and pelvic pain symptoms of CP/CPPS patients.

### 3.4 Abnormal gray matter volume and density in CP/CPPS patients after treatment compared with HCs

**Figure 3:**
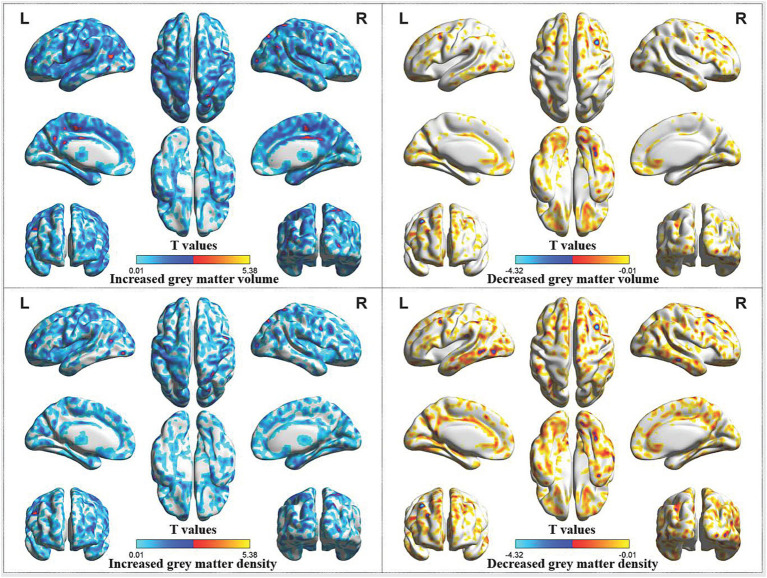
Abnormal gray matter volume and density in CP/CPPS patients after treatment compared with HCs. CP/CPPS, chronic prostatitis/chronic pelvic pain syndrome; HCs, healthy controls; L, left; R, right.

Compared with HCs, CP/CPPS patients after treatment had increased gray matter volume in the left middle cingulate gyrus, left precentral gyrus, left middle temporal gyrus, left middle occipital gyrus, left putamen, right middle cingulate gyrus, right superior parietal gyrus, right postcentral gyrus, right parahippocampal gyrus, as well as decreased gray matter volume in the right middle frontal gyrus. Moreover, patients following treatment showed increased gray matter density in the left precentral gyrus, left inferior occipital gyrus and decreased gray matter density in the right middle frontal gyrus, right middle occipital gyrus (Table 1; Figure 3).

Among all these results, decreased gray matter volume and gray matter density in the right middle frontal gyrus, as well as decreased gray matter density in the right middle occipital gyrus, might be related to the therapeutic effect of acupuncture, which was achieved by inhibiting compensatory increase in gray matter structure in the frontal and occipital lobes of CP/CPPS patients before treatment.

### 3.5 Abnormal white matter volume and density in CP/CPPS patients after treatment compared with HCs

**Figure 4:**
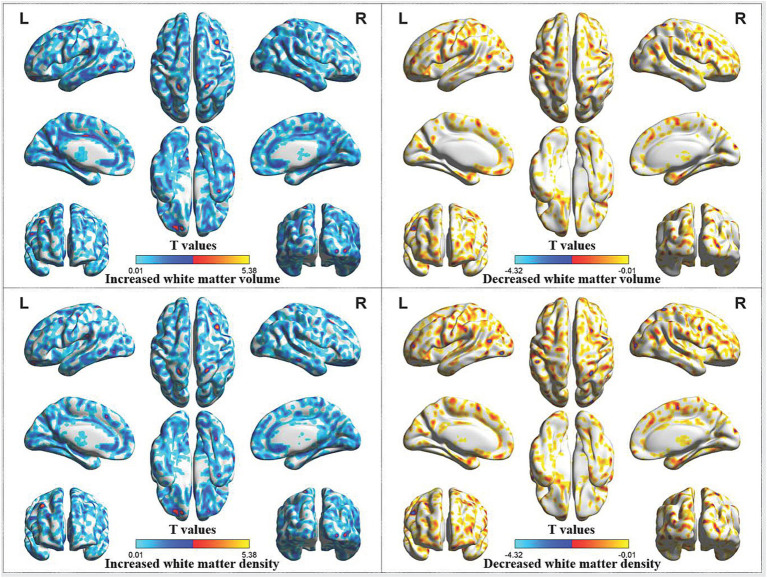
Abnormal white matter volume and density in CP/CPPS patients after treatment compared with HCs. CP/CPPS, chronic prostatitis/chronic pelvic pain syndrome; HCs, healthy controls; L, left; R, right.

Compared with HCs, CP/CPPS patients after treatment demonstrated increased white matter volume in the left orbital superior frontal gyrus, left medial superior frontal gyrus, left superior parietal gyrus, left supramarginal gyrus, right middle frontal gyrus, right postcentral gyrus, right middle temporal gyrus, right middle occipital gyrus and right angular gyrus. Additionally, these patients showed increased white matter density in the left orbital superior frontal gyrus, left superior parietal gyrus, left postcentral gyrus, left supramarginal gyrus, right postcentral gyrus, right middle frontal gyrus, right middle occipital gyrus and decreased white matter density in the left precentral gyrus, left middle occipital gyrus (Table 2; Figure 4).

Decreased white matter density in the left precentral gyrus, left middle occipital gyrus, might be also related to the therapeutic mechanism of acupuncture in improving clinical symptoms in CP/CPPS patients. Acupuncture might achieve therapeutic effects by relieving compensatory increases in white matter in the frontal and occipital lobes of CP/CPPS patients.

### 3.6 Changes of brain structure in CP/CPPS patients after and before treatment

**Figure 5:**
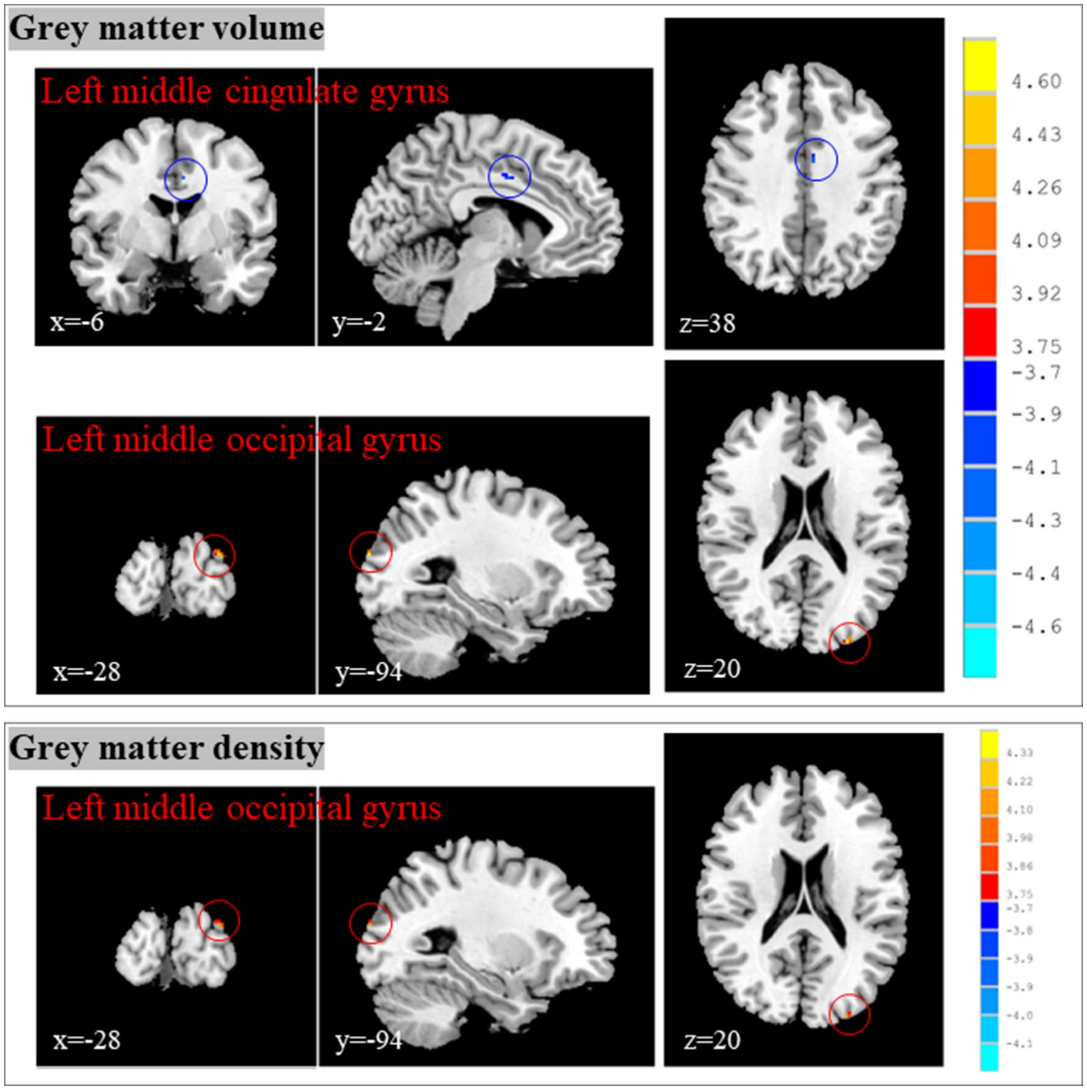
Changes of brain structure in CP/CPPS patients after and before treatment. CP/CPPS, chronic prostatitis/chronic pelvic pain syndrome; HCs, healthy controls; MNI, Montreal Neurological Institute; x, y, and z: the coordinates of peak voxels of clusters in MNI space.

Compared with the brain structure before treatment, CP/CPPS patients showed decreased gray matter volume in the left middle cingulate gyrus, as well as increased gray matter volume and density in the left middle occipital gyrus after treatment (Table 1; Figure 5).

The middle cingulate cortex is an important functional zone of the cingulate cortex, and its core functions can be summarized as follows: motor control and coordination, visceral function regulation, cognitive and emotional integration. Therefore, the most important structural change among these brain regions was the decreased gray matter volume in the left middle cingulate gyrus. Changes of brain structure in CP/CPPS patients after and before treatment suggested that the effects of acupuncture in improving clinical symptoms of CP/CPPS might be achieved by reducing the gray matter volume in the left middle cingulate gyrus.

### 3.7 Associations between NIH-CPSI scores and brain structure in CP/CPPS patients

**Figure 6:**
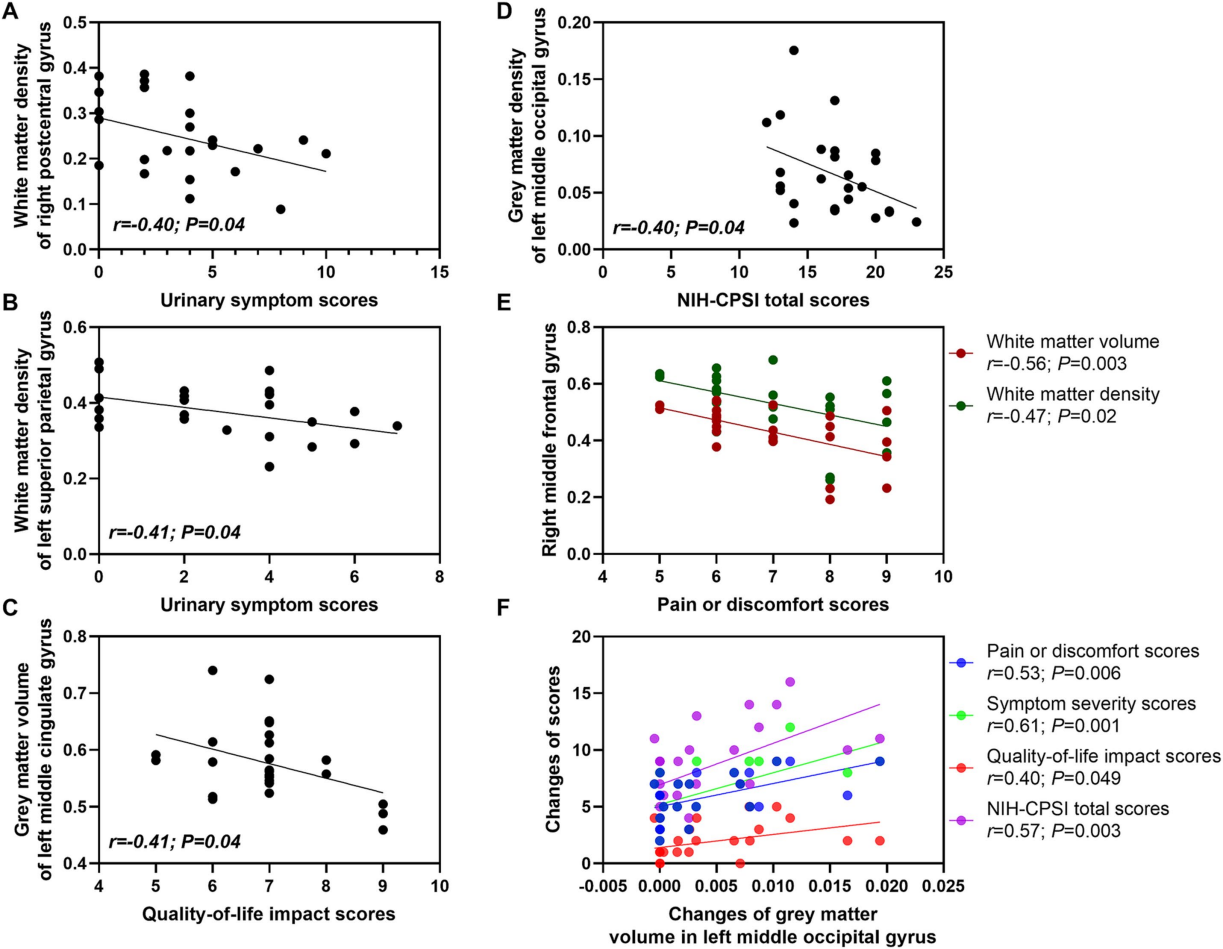
Associations between NIH-CPSI scores and brain structure in CP/CPPS patients. CP/CPPS, chronic prostatitis/chronic pelvic pain syndrome; NIH-CPSI, National Institute of Health-Chronic Prostatitis Symptom Index. **A–E**, associations between NIH-CPSI scores and brain structure in CP/CPPS patients before **(A)** and after **(B–E)** treatment. **(F)** Associations between changes of NIH-CPSI scores and changes of brain structure in CP/CPPS patients. *p* value was obtained by *Pearson* correlation analysis. *p* < 0.05 was considered to be statistically significant difference.

In CP/CPPS patients before treatment, the urinary symptom scores were negatively associated with the white matter density of right postcentral gyrus (*r* = −0.40; *p* = 0.04) (Figure 6).

In CP/CPPS patients after treatment, the pain or discomfort scores were negatively associated with the white matter volume of right middle frontal gyrus (*r* = −0.56; *p* = 0.003) and the white matter density of right middle frontal gyrus (*r* = −0.47; *p* = 0.02). The urinary symptom scores were negatively associated with the white matter density of left superior parietal gyrus (*r* = −0.41; *p* = 0.04). The quality-of-life impact scores were negatively associated with the gray matter volume of left middle cingulate gyrus (*r* = −0.41; *p* = 0.04). The NIH-CPSI total scores were negatively associated with the gray matter density of left middle occipital gyrus (*r* = −0.40; *p* = 0.04) (Figure 6).

The changes of gray matter volume in the left middle occipital gyrus were positively associated with the changes of pain or discomfort scores (*r* = 0.53; *p* = 0.006), symptom severity scores (*r* = 0.61; *p* = 0.001), quality-of-life impact scores (*r* = 0.40; *p* = 0.049) and NIH-CPSI total scores (*r* = 0.57; *p* = 0.003) (Figure 6).

These correlation analysis results further revealed the brain regions that might be related to the development of CP/CPPS, as well as the mechanism of acupuncture improving clinical symptoms of CPPS patients. These findings suggested that the development of CP/CPPS might be associated with the increased gray matter and white matter in the frontal, cingulate and parietal regions. The effects of acupuncture in improving clinical symptoms of CP/CPPS might be achieved by reducing the gray matter volume in the left middle cingulate gyrus (Table 3).

**Table 3:** Brain regions exhibited abnormal white matter volume and density in CP/CPPS patients.

Brain regions	Peak MNI coordinates			Clusters	Peak *t* values
	*x*	*y*	*z*		
White matter volume					
Patients before treatment vs HCs					
Left orbital superior frontal gyrus	−16	50	−18	8	4.0.42
Left medial superior frontal gyrus	−10	22	42	12	4.62
Left superior parietal gyrus	−20	−46	66	21	4.69
Left postcentral gyrus	−52	−14	34	7	4.71
Left inferior temporal gyrus	−50	−20	−30	7	4.33
Left supramarginal gyrus	−50	−28	24	19	5.32
Right middle frontal gyrus	34	20	44	29	4.41
Right postcentral gyrus	22	−44	68	22	4.95
Right middle temporal gyrus	62	−28	−14	10	3.91
Right middle occipital gyrus	36	−78	2	19	4.60
Right angular gyrus	42	−48	32	17	4.62
Patients after treatment vs HCs					
Left orbital superior frontal gyrus	−16	50	−18	7	4.22
Left medial superior frontal gyrus	−10	22	42	10	4.55
Left superior parietal gyrus	−20	−46	64	19	4.69
Left supramarginal gyrus	−50	−28	24	14	4.93
Right middle frontal gyrus	34	20	44	26	4.33
Right postcentral gyrus	22	−44	68	19	4.73
Right middle temporal gyrus	60	−30	−12	10	3.89
Right middle occipital gyrus	36	−78	2	20	4.56
Right angular gyrus	42	−48	32	15	4.57
Patients after vs before treatment					
No significant differences					
White matter density					
Patients before treatment vs HCs					
Left orbital superior frontal gyrus	−16	50	−18	12	4.60
Left superior parietal gyrus	−20	−46	64	16	4.43
Left postcentral gyrus	−52	−14	34	9	4.44
Left supramarginal gyrus	−50	−28	24	10	4.66
Left precentral gyrus	−36	2	44	8	−5.37
Right middle frontal gyrus	36	20	44	29	4.41
Right middle occipital gyrus	36	−78	2	19	4.62
Right postcentral gyrus	22	−44	68	16	4.58
Patients after treatment vs HCs					
Left orbital superior frontal gyrus	−16	50	−18	9	4.43
Left superior parietal gyrus	−20	−46	64	18	4.51
Left postcentral gyrus	−52	−14	34	7	4.30
Left supramarginal gyrus	−50	−28	24	8	4.39
Left precentral gyrus	−36	2	44	9	−5.30
Left middle occipital gyrus	−42	−82	−2	8	−4.12
Right postcentral gyrus	22	−44	68	14	4.40
Right middle frontal gyrus	34	20	44	28	4.36
Right middle occipital gyrus	36	−78	2	19	4.62
Patients after vs before treatment					
No significant differences					

CP/CPPS, chronic prostatitis/chronic pelvic pain syndrome; MNI, Montreal Neurological Institute; x, y, and z, the coordinates of peak voxels of clusters in MNI space. The significant difference was set at *P* < 0.001 and the minimum cluster size was 6 voxels, which was corrected by the AlphaSim program in REST software.

## 4 Discussion

In this study, the findings showed that (1) CP/CPPS patients demonstrated decreased scores in the scale of NIH-CPSI and its subscales after acupuncture; (2) both CP/CPPS patients before and after treatment exhibited increased volume and density of gray matter and white matter, especially in the frontal, cingulate and parietal regions compared with HCs; (3) CP/CPPS patients displayed decreased gray matter volume in the left middle cingulate gyrus, as well as increased gray matter volume and density in the left middle occipital gyrus after treatment; (4) altered gray matter and white matter were associated with CP/CPPS symptoms and related to the mechanisms of acupuncture in treating CP/CPPS.

Acupuncture, a critical component of TCM, can be used for improving CP/CPPS symptoms and reduce pain (44). The mechanism of acupuncture for CP/CPPS is mostly achieved by modulating the associated neurotransmitters, regulating the effects of inflammatory factors, or modulating immune responses in patients or models with CP/CPPS (45). Systematic review and meta-analysis study showed that acupuncture could achieve superior effects in improving pains, urinary symptom, quality of life, NIH-CPSI score, and efficacy than western medicine and sham acupuncture (30). In this study, decreased scores in the scale of NIH-CPSI and its subscales were found in CP/CPPS patients after acupuncture, which were consistent with previous studies. However, the mechanisms, especially the central mechanisms associated with acupuncture in treating CP/CPPS are still unknown. From the insight of peripheral systems, acupuncture can reduce the expression level of inflammatory factors, increase the expression levels of anti-inflammatory factors, which enhance the immune function of patients (46). Oxidative stress was considered as a major pathway involved in the occurrence of chronic prostatitis, and the oxidative stress cascade was regard as a potential target for the treatment of prostatic diseases (47). The release of substance P and the inhibition of β-endorphin secreted by immune cells might may account for the persistence of pain symptoms (48). Acupuncture can be used for alleviating the pain symptoms of CP/CPPS patients by the aforementioned mechanisms (49). In addition, the central analgesic effect of acupuncture was mainly achieved by inhibiting the formation of synaptic plasticity, reducing the sensitivity of central nervous system and inhibiting glial cell activation (50). Moreover, the persistent chronic pain of CP/CPPS patients could lead to decreased activity and gray matter volume in the anterior cingulate gyrus of the brain, which were correlated with the NIH-CPSI total score and pain subscale (51). Therefore, there was an urgent need for identifying changes of brain structure associated with acupuncture in alleviating CP/CPPS symptoms.

Previous structural neuroimaging studies showed abnormal gray matter and white matter in pain-transmitting areas in patients suffering from chronic pain (52–54). The local alterations of brain structure were related to the transmission of pain and were considered as the consequence of pain (55). These changes of brain morphology could be normalized when pain is successfully treated, which suggested that brain structural abnormalities might be a reversible consequence of chronic nociceptive transmission (55). In this study, CP/CPPS patients before acupuncture showed increased gray matter volume in the middle cingulate gyrus, precentral gyrus, superior parietal gyrus, middle temporal gyrus, middle occipital gyrus, increased gray matter density in the precentral gyrus, decreased gray matter volume in the middle frontal gyrus, and decreased gray matter density in the middle frontal gyrus and middle occipital gyrus when compared with HCs. Moreover, patients before acupuncture also exhibited increased white matter volume in the superior frontal gyrus, superior parietal gyrus, postcentral gyrus, middle and inferior temporal gyrus, supramarginal gyrus, middle frontal gyrus, middle occipital gyrus and angular gyrus, increased white matter density in the superior frontal gyrus, superior parietal gyrus, postcentral gyrus, supramarginal gyrus, middle frontal gyrus, middle occipital gyrus, postcentral gyrus, and decreased white matter density in the precentral gyrus. Previous MRI study demonstrated that the spontaneous pelvic pain of CP/CPPS patients was associated with the activation within the right anterior insula, while the density of gray matter in anterior insula and anterior cingulate cortices were correlated with the pain intensity and extent of pain chronicity (56). In addition, these CP/CPPS patients exhibited impaired relationships between white matter anisotropy and neocortical gray matter volume. The anatomical changes in the cingulate cortices were associated with the unique ongoing experience of pelvic pain and CP/CPPS patients with greater pain likely had increased gray matter density in the cingulate cortices. In this study, we found that CP/CPPS patients before treatment exhibited increased volume and density of gray matter and white matter in widespread brain regions, especially in the frontal, cingulate and parietal cortices, as well as slight decline of gray matter in the middle frontal gyrus. In our previous study (8), we also found that CP/CPPS had higher global efficiency in the left middle cingulate and paracingulate gyrus, and increased local efficiency in the left middle cingulate, paracingulate gyrus and paracentral lobule. Females with chronic pelvic pain presented increased functional connectivity between the cingulate cortex and other brain regions involved in the processes and regulation of pain, sensory, motor and emotion (57). In addition, the altered functional connectivity in the cingulate cortex was associated with phenotype measures of these patients, which included pain and urologic symptom intensity, depression, anxiety, quality of relationships and self-esteem levels. All these findings implied that abnormal function in the cingulate cortex might lead to impaired neurological processes of self-referential thought and introspection, which then resulted in pain and negative emotion in patients with urologic chronic pelvic pain syndrome.

The brain regions including anterior cingulate gyrus, postcentral gyrus, precuneus and insula were related to pain feelings of CP/CPPS patients, which could also lead to dysregulation of painful emotions, lowering the tolerance of patients to stimulus (11). CP/CPPS patients showed augmented brain activity in the inferior parietal lobule and cingulate gyrus when compared with HCs, which might play an important role in the pathogenesis of CP/CPPS. However, CP/CPPS patients exhibited decreased consistency of activities in the bilateral anterior cingulate cortices and right medial prefrontal cortex involved in the pain modulation process when compared with HCs, which were correlated with the NIH-CPSI total score and pain subscale (58). Therefore, the increased function or structure in the cingulate might be a compensatory coping strategy in chronic pain condition, which might be associated with aberrant central control in pain processing (an impaired pain modulatory system, either by decreased descending pain inhibition or enhanced pain facilitation) in CP/CPPS patients (59). In neuropathic pain mice, increased activity of pyramidal cells in the cingulate cortex caused impaired excitatory/inhibitory balance, which exacerbated pain hypersensitivity (60). The brain regions including the cingulate cortex, involved in emotion-related behaviors including pain, were highly plastic, and modulating their structural or functional plasticity of synapses could inhibit central sensitization and thus reduce neuropathic pain (61, 62). The inhibition of the cingulate cortex could relieve pain in the rodent model of chronic pain (63). In this study, CP/CPPS patients showed decreased gray matter volume in the left middle cingulate gyrus after acupuncture. Acupuncture provoked extensive changes in brain function and the cingulate cortex was selected as the hub region after acupuncture, which also suggested that peripheral ERK signaling induced by acupuncture played a key role in modulating acupuncture-induced brain neural activity (64). Acupuncture could alleviate pain affect by using information integration from the autonomic brain regions including the cingulate and frontal cortex, key parts of the cortical representation of pain (65). Previous fMRI study exploring the regulatory effect of acupuncture on the brain showed that the functional connectivity of the cingulate cortex was decreased in the patients after acupuncture, which implied that acupuncture could effectively relieve the clinical symptoms of patients, and promote the functional re-construction (66). Moreover, acupuncture could block excessive stimulation of abnormal pain signals in the brain and spinal cord by inhibiting the activation of N-methyl-D-aspartate receptor ion channels in the cingulate cortex or releasing opioids (67). Based on aforementioned studies, we speculated that the effects of acupuncture in improving clinical symptoms of CP/CPPS might be achieved by reducing the gray matter volume in the left middle cingulate gyrus.

In CP/CPPS patients before treatment, the urinary symptom scores were negatively associated with the white matter density of right postcentral gyrus. The postcentral gyrus is an important sensory center in the cerebral cortex, with the following main functions: sensory information processing (receiving sensory signals, analyzing and integrating information), sensory localization and correspondence (fine sensory processing), and participation in sensory related functions (entity perception, motor perception). Therefore, the abnormalities of white matter in the right postcentral gyrus might be associated with LUTS symptoms of CP/CPPS patients. In addition, the pain or discomfort scores, urinary symptom scores, quality-of-life impact scores and NIH-CPSI total scores were mainly associated with abnormal structures in the frontal, cingulate and parietal regions of CP/CPPS patients after treatment. The frontal–parietal network of the brain has multiple important functions, mainly including cognitive control (task initiation and switching, flexible regulation), attention allocation, working memory (information storage, information retention and processing), somatosensory perception and pain processing. Therefore, abnormal structures in the frontal, cingulate and parietal regions might be related to various clinical symptoms, even in CP/CPPS patients after acupuncture treatment. Finally, the changes of gray matter volume in the left middle occipital gyrus were positively associated with the changes of pain or discomfort scores, symptom severity scores, quality-of-life impact scores and NIH-CPSI total scores. These findings suggested that the effects of acupuncture in improving clinical symptoms of CP/CPPS might be achieved by reducing the gray matter volume in the left middle cingulate gyrus.

However, several limitations should be addressed in this study. Firstly, the sample size of this study was relatively small and larger cohort studies were needed. Secondly, considering that there was no unified standard for sham acupuncture, the current study did not include a sham acupuncture control group. Therefore, the lack of a sham acupuncture control group was considered another limitation. However, with the deepening of future research findings and the continuous progress of research methods, the sham acupuncture control study would be further used to verify the results of the current study. Thirdly, the efficacy and brain modulation mechanisms of acupuncture for CP/CPPS at different time points were not explored. Therefore, further longitudinal follow-up studies with larger sample size were needed to evaluate the short term and long-term effects of acupuncture, as well as the underlying central neural mechanisms.

## 5 Conclusion

To the best of our knowledge, this was the first structural MRI study exploring the efficacy of acupuncture and its brain modulation mechanisms for CP/CPPS. The findings prompted that the development of CP/CPPS might be related to increased volume and density of gray matter and white matter in the frontal, cingulate and parietal regions. Additionally, acupuncture showed good therapeutic effects in improving CP/CPPS symptoms by decreasing gray matter volume in the left middle cingulate gyrus. All these provided new insights into the brain modulation mechanisms of acupuncture for CP/CPPS from the view of neuroimaging.

## Data Availability

The raw data supporting the conclusions of this article will be made available by the authors, without undue reservation.

## References

[1] Mehik A Hellström P Lukkarinen O Sarpola A Järvelin M. Epidemiology of prostatitis in Finnish men: a population-based cross-sectional study. BJU Int. (2000) 86:443–8. doi: 10.1046/j.1464-410x.2000.00836.x, PMID: 10971269

[2] Franco JV Turk T Jung JH Xiao YT Iakhno S Tirapegui FI. Pharmacological interventions for treating chronic prostatitis/chronic pelvic pain syndrome. Cochrane Database Syst Rev. (2019) 10:Cd012552. doi: 10.1002/14651858.CD012552.pub2, PMID: 31587256 PMC6778620

[3] Nickel JC Nyberg LM Hennenfent M. Research guidelines for chronic prostatitis: consensus report from the first National Institutes of Health international prostatitis collaborative network. Urology. (1999) 54:229–33. doi: 10.1016/s0090-4295(99)00205-8, PMID: 10443716

[4] Krieger JN Berger RE Ross SO Rothman I Muller CH. Seminal fluid findings in men with nonbacterial prostatitis and prostatodynia. J Androl. (1996) 17:310–8. doi: 10.1002/j.1939-4640.1996.tb01788.x, PMID: 8792222

[5] Nickel JC Alexander R Anderson R Krieger J Moon T Neal D. Prostatitis unplugged? Prostatic massage revisited. Tech Urol. (1999) 5:1–7. 10374787

[6] Rees J Abrahams M Doble A Cooper A. Diagnosis and treatment of chronic bacterial prostatitis and chronic prostatitis/chronic pelvic pain syndrome: a consensus guideline. BJU Int. (2015) 116:509–25. doi: 10.1111/bju.13101, PMID: 25711488 PMC5008168

[7] Huang X Qin Z Cui H Chen J Liu T Zhu Y. Psychological factors and pain catastrophizing in men with chronic prostatitis/chronic pelvic pain syndrome (CP/CPPS): a meta-analysis. Transl Androl Urol. (2020) 9:485–93. doi: 10.21037/tau.2020.01.25, PMID: 32420154 PMC7214995

[8] Huang X Chen J Liu S Gong Q Liu T Lu C. Impaired frontal-parietal control network in chronic prostatitis/chronic pelvic pain syndrome revealed by graph theoretical analysis: a DTI study. Eur J Neurosci. (2021) 53:1060–71. doi: 10.1111/ejn.14962, PMID: 32896914

[9] He H Luo H Qian B Xu H Zhang G Zou X. Autonomic nervous system dysfunction is related to chronic prostatitis/chronic pelvic pain syndrome. World J Men’s Health. (2024) 42:1–28. doi: 10.5534/wjmh.220248, PMID: 37118962 PMC10782122

[10] Chen L Bian Z Chen J Meng J Zhang M Liang C. Immunological alterations in patients with chronic prostatitis/chronic pelvic pain syndrome and experimental autoimmune prostatitis model: a systematic review and meta-analysis. Cytokine. (2021) 141:155440. doi: 10.1016/j.cyto.2021.155440, PMID: 33550164

[11] Zhao Y Lin J Dong Y Tian Z Ye Y Ma Z. Neuroimaging studies of chronic prostatitis/chronic pelvic pain syndrome. Pain Res Manag. (2022) 2022:9448620. doi: 10.1155/2022/9448620, PMID: 35573644 PMC9095382

[12] Pontari MA. Etiology of chronic prostatitis/chronic pelvic pain syndrome: psychoimmunoneurendocrine dysfunction (PINE syndrome) or just a really bad infection? World J Urol. (2013) 31:725–32. doi: 10.1007/s00345-013-1061-z, PMID: 23579440

[13] Clemens JQ Mullins C Ackerman AL Bavendam T van Bokhoven A Ellingson BM. Urologic chronic pelvic pain syndrome: insights from the MAPP research network. Nat Rev Urol. (2019) 16:187–200. doi: 10.1038/s41585-018-0135-5, PMID: 30560936 PMC6800057

[14] Krsmanovic A Tripp DA Nickel JC Shoskes DA Pontari M Litwin MS. Psychosocial mechanisms of the pain and quality of life relationship for chronic prostatitis/chronic pelvic pain syndrome (CP/CPPS). Can Urol Assoc J. (2014) 8:403–8. doi: 10.5489/cuaj.2179, PMID: 25553153 PMC4277519

[15] Tripp DA Nickel JC Shoskes D Koljuskov A. A 2-year follow-up of quality of life, pain, and psychosocial factors in patients with chronic prostatitis/chronic pelvic pain syndrome and their spouses. World J Urol. (2013) 31:733–9. doi: 10.1007/s00345-013-1067-6, PMID: 23568443

[16] Reddan MC Wager TD. Brain systems at the intersection of chronic pain and self-regulation. Neurosci Lett. (2019) 702:24–33. doi: 10.1016/j.neulet.2018.11.047, PMID: 30503923

[17] Malfliet A Coppieters I Van Wilgen P Kregel J De Pauw R Dolphens M. Brain changes associated with cognitive and emotional factors in chronic pain: a systematic review. Eur J Pain (London, England). (2017) 21:769–86. doi: 10.1002/ejp.1003, PMID: 28146315

[18] Mercer Lindsay N Chen C Gilam G Mackey S Scherrer G. Brain circuits for pain and its treatment. Sci Transl Med. (2021) 13:eabj7360. doi: 10.1126/scitranslmed.abj7360, PMID: 34757810 PMC8675872

[19] Ng SK Urquhart DM Fitzgerald PB Cicuttini FM Hussain SM Fitzgibbon BM. The relationship between structural and functional brain changes and altered emotion and cognition in chronic low Back pain brain changes: a systematic review of MRI and fMRI studies. Clin J Pain. (2018) 34:237–61. doi: 10.1097/ajp.0000000000000534, PMID: 28719509

[20] Yang S Chang MC. Chronic pain: structural and functional changes in brain structures and associated negative affective states. Int J Mol Sci. (2019) 20:3130. doi: 10.3390/ijms20133130, PMID: 31248061 PMC6650904

[21] Lan X Zhu XY Bai WX Liu HP Wang H Dun WH. White matter changes in young and middle-aged males with chronic prostatitis/chronic pelvic pain syndrome: tract-based spatial statistics analysis. Eur J Neurosci. (2023) 58:3892–902. doi: 10.1111/ejn.16154, PMID: 37779210

[22] Ge S Hu Q Guo Y Xu K Xia G Sun C. Potential alterations of functional connectivity analysis in the patients with chronic prostatitis/chronic pelvic pain syndrome. Neural Plast. (2021) 2021:1–9. doi: 10.1155/2021/6690414, PMID: 34035803 PMC8121565

[23] Kutch JJ Yani MS Asavasopon S Kirages DJ Rana M Cosand L. Altered resting state neuromotor connectivity in men with chronic prostatitis/chronic pelvic pain syndrome: a MAPP: research network neuroimaging study. NeuroImage Clin. (2015) 8:493–502. doi: 10.1016/j.nicl.2015.05.013, PMID: 26106574 PMC4474411

[24] Pena VN Engel N Gabrielson AT Rabinowitz MJ Herati AS. Diagnostic and management strategies for patients with chronic prostatitis and chronic pelvic pain syndrome. Drugs Aging. (2021) 38:845–86. doi: 10.1007/s40266-021-00890-2, PMID: 34586623

[25] Polackwich AS Shoskes DA. Chronic prostatitis/chronic pelvic pain syndrome: a review of evaluation and therapy. Prostate Cancer Prostatic Dis. (2016) 19:132–8. doi: 10.1038/pcan.2016.8, PMID: 26951713

[26] Cohen JM Fagin AP Hariton E Niska JR Pierce MW Kuriyama A. Therapeutic intervention for chronic prostatitis/chronic pelvic pain syndrome (CP/CPPS): a systematic review and meta-analysis. PloS one. (2012) 7:e41941. doi: 10.1371/journal.pone.0041941, PMID: 22870266 PMC3411608

[27] Franco JV Turk T Jung JH Xiao YT Iakhno S Garrote V. Non-pharmacological interventions for treating chronic prostatitis/chronic pelvic pain syndrome. Cochrane Database Syst Rev. (2018) 2018:Cd012551. doi: 10.1002/14651858.CD012551.pub3, PMID: 29757454 PMC6494451

[28] EAU Guidelines. EAU Guidelines. Edn. Presented at the EAU annual congress. Madrid: (2025) ISBN 978-94-92671-29-5.

[29] Qin Z Guo J Chen H Wu J. Acupuncture for chronic prostatitis/chronic pelvic pain syndrome: a GRADE-assessed systematic review and Meta-analysis. Eur Urol Open Sci. (2022) 46:55–67. doi: 10.1016/j.euros.2022.10.005, PMID: 36506258 PMC9732484

[30] Pan J Jin S Xie Q Wang Y Wu Z Sun J. Acupuncture for chronic prostatitis or chronic pelvic pain syndrome: an updated systematic review and meta-analysis. Pain Res Manag. (2023) 2023:7754876. doi: 10.1155/2023/7754876, PMID: 36960418 PMC10030225

[31] Cai RL Shen GM Wang H Guan YY. Brain functional connectivity network studies of acupuncture: a systematic review on resting-state fMRI. J Integr Med. (2018) 16:26–33. doi: 10.1016/j.joim.2017.12.002, PMID: 29397089

[32] Huang H Yue X Huang X Long W Kang S Rao Y. Brain activities responding to acupuncture at ST36 (zusanli) in healthy subjects: a systematic review and Meta-analysis of task-based fMRI studies. Front Neurol. (2022) 13:930753. doi: 10.3389/fneur.2022.930753, PMID: 35968313 PMC9373901

[33] Liu L Lyu TL Fu MY Wang LP Chen Y Hong JH. Changes in brain connectivity linked to multisensory processing of pain modulation in migraine with acupuncture treatment. NeuroImage Clin. (2022) 36:103168. doi: 10.1016/j.nicl.2022.103168, PMID: 36067612 PMC9468576

[34] Krieger JN Nyberg L Jr Nickel JC. NIH consensus definition and classification of prostatitis. JAMA. (1999) 282:236–7. doi: 10.1001/jama.282.3.236, PMID: 10422990

[35] Litwin MS McNaughton-Collins M Fowler FJ Jr Nickel JC Calhoun EA Pontari MA. The National Institutes of Health chronic prostatitis symptom index: development and validation of a new outcome measure. Chronic Prostatitis Collaborative Research Network. J Urol. (1999) 162:369–75. doi: 10.1016/s0022-5347(05)68562-x, PMID: 10411041

[36] Gao S Chen J Liu J Guan Y Liu R Yang J. Decreased grey matter volume in dorsolateral prefrontal cortex and thalamus accompanied by compensatory increases in middle cingulate gyrus of premature ejaculation patients. Andrology. (2024) 12:841–9. doi: 10.1111/andr.13547, PMID: 37902180

[37] Chao-Gan Y Yu-Feng Z. DPARSF: a MATLAB toolbox for "pipeline" data analysis of resting-state fMRI. Front Syst Neurosci. (2010) 4:13. doi: 10.3389/fnsys.2010.00013, PMID: 20577591 PMC2889691

[38] Yankowitz LD Yerys BE Herrington JD Pandey J Schultz RT. Dissociating regional gray matter density and gray matter volume in autism spectrum condition. NeuroImage Clin. (2021) 32:102888. doi: 10.1016/j.nicl.2021.102888, PMID: 34911194 PMC8633367

[39] Liu H Lin J Shang H. Voxel-based meta-analysis of gray matter and white matter changes in patients with spinocerebellar ataxia type 3. Front Neurol. (2023) 14:1197822. doi: 10.3389/fneur.2023.1197822, PMID: 37576018 PMC10413272

[40] Uhlmann A Dias A Taljaard L Stein DJ Brooks SJ Lochner C. White matter volume alterations in hair-pulling disorder (trichotillomania). Brain Imaging Behav. (2020) 14:2202–9. doi: 10.1007/s11682-019-00170-z, PMID: 31376114

[41] Howes OD Cummings C Chapman GE Shatalina E. Neuroimaging in schizophrenia: an overview of findings and their implications for synaptic changes. Neuropsychopharmacology. (2023) 48:151–67. doi: 10.1038/s41386-022-01426-x, PMID: 36056106 PMC9700830

[42] Yu H Meng YJ Li XJ Zhang C Liang S Li ML. Common and distinct patterns of grey matter alterations in borderline personality disorder and bipolar disorder: voxel-based meta-analysis. Br J Psychiatry. (2019) 215:395–403. doi: 10.1192/bjp.2019.44, PMID: 30846010

[43] Song XW Dong ZY Long XY Li SF Zuo XN Zhu CZ. REST: a toolkit for resting-state functional magnetic resonance imaging data processing. PLoS One. (2011) 6:e25031. doi: 10.1371/journal.pone.0025031, PMID: 21949842 PMC3176805

[44] Hu J Xiao Y Jiang G Hu X. Research trends of acupuncture therapy on chronic pelvic pain syndrome from 2000 to 2022: a bibliometric analysis. J Pain Res. (2023) 16:4049–69. doi: 10.2147/jpr.S434333, PMID: 38054110 PMC10695139

[45] Wang H Zhang J Ma D Zhao Z. The role of acupuncture and its related mechanism in treating chronic prostatitis/chronic pelvic pain syndrome. Int J Gen Med. (2023) 16:4039–50. doi: 10.2147/ijgm.S417066, PMID: 37700742 PMC10493142

[46] Wazir J Ullah R Li S Hossain MA Diallo MT Khan FU. Efficacy of acupuncture in the treatment of chronic prostatitis-chronic pelvic pain syndrome: a review of the literature. Int Urol Nephrol. (2019) 51:2093–106. doi: 10.1007/s11255-019-02267-2, PMID: 31468287

[47] Roumeguère T Sfeir J El Rassy E Albisinni S Van Antwerpen P Boudjeltia KZ. Oxidative stress and prostatic diseases. Mol Clin Oncol. (2017) 7:723–8. doi: 10.3892/mco.2017.1413, PMID: 29181163 PMC5700279

[48] Shahed AR Shoskes DA. Correlation of beta-endorphin and prostaglandin E2 levels in prostatic fluid of patients with chronic prostatitis with diagnosis and treatment response. J Urol. (2001) 166:1738–41. doi: 10.1016/S0022-5347(05)65664-9, PMID: 11586213

[49] Ma Y Li X Li F Yu W Wang Z. Clinical research of chronic pelvic cavity pain syndrome treated with acupoint catgut embedding therapy. Zhongguo Zhen Jiu. (2015) 35:561–6. 26480553

[50] Wang QY Qu YY Feng CW Sun WB Wang DL Yang TS. Analgesic mechanism of acupuncture on neuropathic pain. Zhongguo Zhen Jiu. (2020) 40:907–12. doi: 10.13703/j.0255-2930.20190927-k0003 32869605

[51] Mordasini L Weisstanner C Rummel C Thalmann GN Verma RK Wiest R. Chronic pelvic pain syndrome in men is associated with reduction of relative gray matter volume in the anterior cingulate cortex compared to healthy controls. J Urol. (2012) 188:2233–7. doi: 10.1016/j.juro.2012.08.043, PMID: 23083652

[52] Wang Z Yuan M Xiao J Chen L Guo X Dou Y. Gray matter abnormalities in patients with chronic primary pain: a coordinate-based meta-analysis. Pain Physician. (2022) 25:1–13. 35051138

[53] Barroso J Vigotsky AD Branco P Reis AM Schnitzer TJ Galhardo V. Brain gray matter abnormalities in osteoarthritis pain: a cross-sectional evaluation. Pain. (2020) 161:2167–78. doi: 10.1097/j.pain.0000000000001904, PMID: 32379222 PMC7745790

[54] Zhang Y Qu M Yi X Zhuo P Tang J Chen X. Sensorimotor and pain-related alterations of the gray matter and white matter in type 2 diabetic patients with peripheral neuropathy. Hum Brain Mapp. (2020) 41:710–25. doi: 10.1002/hbm.24834, PMID: 31663232 PMC7268085

[55] Rodriguez-Raecke R Niemeier A Ihle K Ruether W May A. Brain gray matter decrease in chronic pain is the consequence and not the cause of pain. J Neurosci Off J Soc Neurosci. (2009) 29:13746–50. doi: 10.1523/jneurosci.3687-09.2009, PMID: 19889986 PMC6666725

[56] Farmer MA Chanda ML Parks EL Baliki MN Apkarian AV Schaeffer AJ. Brain functional and anatomical changes in chronic prostatitis/chronic pelvic pain syndrome. J Urol. (2011) 186:117–24. doi: 10.1016/j.juro.2011.03.027, PMID: 21571326 PMC4889821

[57] Martucci KT Shirer WR Bagarinao E Johnson KA Farmer MA Labus JS. The posterior medial cortex in urologic chronic pelvic pain syndrome: detachment from default mode network-a resting-state study from the MAPP research network. Pain. (2015) 156:1755–64. doi: 10.1097/j.pain.0000000000000238, PMID: 26010458 PMC4545714

[58] Lin Y Bai Y Liu P Yang X Qin W Gu J. Alterations in regional homogeneity of resting-state cerebral activity in patients with chronic prostatitis/chronic pelvic pain syndrome. PLoS One. (2017) 12:e0184896. doi: 10.1371/journal.pone.0184896, PMID: 28926645 PMC5605002

[59] Lan X Niu X Bai WX Li HN Zhu XY Ma WJ. The functional connectivity of the basal ganglia subregions changed in mid-aged and young males with chronic prostatitis/chronic pelvic pain syndrome. Front Hum Neurosci. (2022) 16:1013425. doi: 10.3389/fnhum.2022.1013425, PMID: 36248695 PMC9563619

[60] Zhu DY Cao TT Fan HW Zhang MZ Duan HK Li J. The increased *in vivo* firing of pyramidal cells but not interneurons in the anterior cingulate cortex after neuropathic pain. Mol Brain. (2022) 15:12. doi: 10.1186/s13041-022-00897-9, PMID: 35093140 PMC8800281

[61] Zhuo M. Cortical plasticity as synaptic mechanism for chronic pain. J Neural Transm Vienna, Austria: 1996. (2020) 127:567–73. doi: 10.1007/s00702-019-02071-3, PMID: 31493094

[62] Ge J Cai Y Pan ZZ. Synaptic plasticity in two cell types of central amygdala for regulation of emotion and pain. Front Cell Neurosci. (2022) 16:997360. doi: 10.3389/fncel.2022.997360, PMID: 36385947 PMC9643269

[63] Zhuang X Huang L Gu Y Wang L Zhang R Zhang M. The anterior cingulate cortex projection to the dorsomedial striatum modulates hyperalgesia in a chronic constriction injury mouse model. Arch Med Sci. (2021) 17:1388–99. doi: 10.5114/aoms.2019.85202, PMID: 34522268 PMC8425248

[64] Park JY Cho SJ Lee SH Ryu Y Jang JH Kim SN. Peripheral ERK modulates acupuncture-induced brain neural activity and its functional connectivity. Sci Rep. (2021) 11:5128. doi: 10.1038/s41598-021-84273-y, PMID: 33664320 PMC7933175

[65] Li YW Li W Wang ST Gong YN Dou BM Lyu ZX. The autonomic nervous system: a potential link to the efficacy of acupuncture. Front Neurosci. (2022) 16:1038945. doi: 10.3389/fnins.2022.1038945, PMID: 36570846 PMC9772996

[66] Zhang Y Zha B Shi H Fan Y Zheng Y Rong Z. Exploring the regulatory effect of acupuncture for Ningshen Tongqiao on the functional connectivity between the anterior cingulate cortex and the whole brain in the patients with subjective tinnitus based on fMRI. Zhongguo Zhen Jiu. (2023) 44:12–8. doi: 10.13703/j.0255-2930.20230628-0003 38191153

[67] Yang Y Wang J Zhang C Guo Y Zhao M Zhang M. The efficacy and neural mechanism of acupuncture therapy in the treatment of visceral hypersensitivity in irritable bowel syndrome. Front Neurosci. (2023) 17:1251470. doi: 10.3389/fnins.2023.1251470, PMID: 37732301 PMC10507180

